# Prevalence of growth hormone deficiency in brain tumor survivors: a systematic review and meta-analysis

**DOI:** 10.1530/EO-25-0025

**Published:** 2025-07-08

**Authors:** Tatiana Tselovalnikova, Ben Ponvilawan, Maria G Pavlova, Cynthia Flanagan, Sunpreet S Rakhra, Betty M Drees

**Affiliations:** ^1^ University of Missouri-Kansas City School of Medicine, Kansas City, Missouri, USA; ^2^ First Moscow State Medical University (Sechenov University), Moscow, Russia; ^3^ St. Luke’s Cancer Institute, Kansas City, Missouri, USA; ^4^ Graduate School of the Stowers Institute for Medical Research, Kansas City, Missouri, USA

**Keywords:** growth hormone deficiency, brain tumor, cranial irradiation, growth hormone

## Abstract

**Objective:**

To evaluate the prevalence of growth hormone deficiency in patients who underwent cranial irradiation for brain tumors.

**Methods:**

Ovid Medline and Embase databases were used for review. Eligible studies were observational studies with brain tumor survivors who had growth hormone function evaluated after treatment at age ≥18 years. Patient data on disease prevalence were pooled using the random-effect, generic inverse variance method. The presence of publication bias was determined by Egger’s test. Mann–Whitney U test, univariate and multivariate linear regression were used to determine the association and effect of covariates and growth hormone peak level.

**Results:**

After screening 3,355 relevant articles, seven studies were included. The pooled result showed that out of 3,489 patients who received radiation for brain tumors, regardless of age at the time of treatment, 50% developed growth hormone deficiency (95% CI 40–60%, *I*^2^ = 83%). Subgroup analysis based on the growth hormone peak level did not show differences between different cutoffs. Univariate linear regression using data from 27 patients showed that age at radiation and the time duration between radiation and the stimulation test (*P* = 0.03 and 0.002, respectively), but not radiation dose or sex, were significantly correlated with growth hormone peak level. After multivariate adjustment, only the time duration between radiation and the stimulation test was associated with decreased growth hormone peak level (*P* = 0.04).

**Conclusions:**

Half of brain tumor survivors develop growth hormone deficiency. A longer duration of follow-up is associated with higher risks of growth hormone deficiency. Lifelong follow-up is essential.

## Introduction

Over the last several decades, childhood cancer incidence has been steadily trending up. Since the 1980s, the global rate has increased from 124.0 to 140.6 per million person-years (e.g., incidence; Noordzij *et al.* 2010, Steliarova-Foucher *et al.* 2017). In children aged 0–14 years, the most common types of cancers were leukemia, central nervous system (CNS) tumors, and lymphomas, respectively, while for those aged 5–19 years, CNS tumors were the most common (Price *et al.* 2024). The success of modern treatment strategies has led to an increase in survival rates. For CNS tumors, 5-year survival rates from 1975–1978 and 2003–2007 increased from 51 to 73% in those children aged 0–4 years and from 61 to 76% in aged 5–14 years, respectively (Smith *et al.* 2014).

Despite the increased survival rates, newer treatment strategies for brain tumors have inevitably carried various long-term complications, including those related to the endocrine system. Cranial irradiation, which is one of the principal components of most brain tumor treatment protocols, increases the risk of hormone deficiencies if the radiation field involves the hypothalamic–pituitary (HP) axis. Growth hormone deficiency (GHD) is the first and most common endocrine condition developing after treatment for brain tumors and can result in different manifestations depending on the age when the deficiency occurs (Shalet *et al.* 1976, Darzy & Shalet 2003, Schmiegelow 2006). When it occurs before the closure of growth plates, GHD can significantly affect linear bone growth and eventually result in short stature. In adulthood, GHD can negatively affect body composition, specifically increased fat and reduced lean body mass, reduced bone mineral density, exercise endurance, quality of life, and increased lipid and glucose metabolism disturbances, ultimately leading to increased overall cardiovascular morbidity. According to the guidelines for GHD in adults, all patients with severe GHD are eligible for treatment with growth hormone supplementation (Growth Hormone Research Society 1998). The risk factors for GHD in patients receiving cranial irradiation for brain tumors in childhood are younger age at the time of radiotherapy, longer follow-up period, and higher dose of cranial irradiation (Rappaport & Brauner 1989, Schmiegelow *et al.* 2000*b*, Toogood 2004).

However, there have been no reports from large cohorts regarding the prevalence of GHD in this patient population. In this systematic review and meta-analysis, we aim to evaluate the prevalence of GHD in patients who underwent cranial irradiation for brain tumors using all available eligible studies published to date.

## Methods

### Data sources and searches

All indexed studies were identified from the Medline and Embase databases from their inception to September 2023. Search terms related to ‘GHD’, ‘cranial irradiation’, and ‘brain tumor’ were utilized, with the comprehensive terms listed in Supplementary Data 1 (see section on Supplementary materials given at the end of the article). The Preferred Reporting Items for Systematic Reviews and Meta-Analyses (PRISMA) guidelines for the systematic review and meta-analysis were reported in Supplementary Data 2.

### Selection criteria

Only original articles in English were included. Eligible studies must be observational studies with brain tumor survivors who had their growth hormone function evaluated after cranial irradiation at age ≥18 years. Each study had to report the total number of patients and cases with GHD, defined at the discretion of the investigators. Two investigators (TT and BP) examined the eligibility of the studies. Conflicts were resolved by the senior investigator (BMD).

### Data extraction

A standardized data collection method was used to obtain the following information: the last name of the first author, the year of publication, the country where the study was conducted, the study design, the total number of patients, the type of brain tumor, the type and dose of cranial irradiation used, the receipt of chemotherapy, age at the time of treatment, age at GHD test, the diagnostic criteria of GHD, the percentage of male patients, and comorbidities.

### Quality assessment

The quality of the included studies was assessed using the Jadad quality assessment scoring system for randomized controlled studies by two investigators (TT and BP) (Jadad *et al.* 1996).

### Data analysis

We used R version 4.3.2 software (Austria) for all statistical analyses. The meta-analysis was conducted with the ‘meta’ version 7.0–0 package. The number of GHD patients and the total number of patients from each study were extracted and combined using the inverse variance method to calculate the pooled incidence of GHD, along with its 95% confidence interval (CI). We implemented the random-effects model due to the high inter-study heterogeneity. Statistical heterogeneity was evaluated using Cochran’s Q test and *I*^2^ statistic, with the *I*^2^ values of 0–25%, 26–50%, 51–75%, and >75% signifying insignificant, low, moderate, and high heterogeneity, respectively (Higgins *et al.* 2003). A sensitivity analysis using studies that evaluated GHD at age more than 18 years, along with subgroup analyses based on the cutoff of GH peak level and the continent of the study, were also performed. The presence of publication bias was determined by Egger’s test. We analyzed the association of GH peak level with various covariates (age at cranial irradiation, time duration between the cranial irradiation and GH function test, sex, and cranial irradiation dose) in two studies (Harrop *et al.* 1976, Heikens *et al.* 1998). The associations between GH peak level and each covariate were first evaluated using univariate linear regression separately, and then a multivariate linear regression model with all covariates was performed.

## Results

### Search results

**Figure 1:**
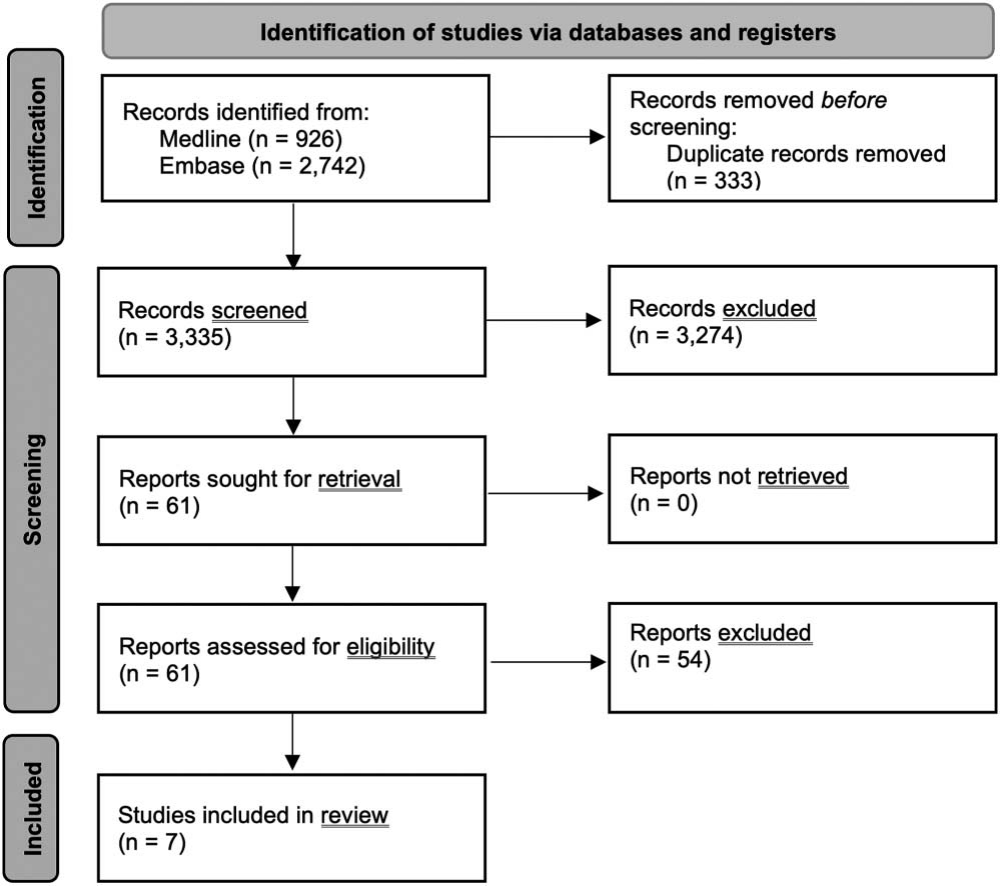
Study identification and the literature review process.

In total, 3,668 articles were retrieved from Medline and Embase databases, of which 333 were duplicates and were removed, leaving 3,335 articles for the title and abstract review. Subsequently, 3,274 articles did not fulfill the inclusion criteria and were discarded, resulting in 61 articles for full-length article review. The second round of review revealed that 54 of them did not report the outcomes of interest and were further excluded. Ultimately, seven cohort studies with 3,510 patients were eligible for this meta-analysis (Harrop *et al.* 1976, Shalet *et al.* 1977, Heikens *et al.* 1998, Popovic *et al.* 2002, van Iersel *et al.* 2019, Maciel *et al.* 2021, Baunsgaard *et al.* 2022). Supplementary Table S1 depicts the characteristics of all included studies. The PRISMA diagram is depicted in Fig. 1.

### Study characteristics

Five studies utilized the insulin tolerance test (ITT) to diagnose GHD, with varying cutoff values. Harrop *et al.* defined a peak GH ≥ 20 mU/L as indicating intact GH secretion (Harrop *et al.* 1976), while Heikens *et al.* considered a peak GH > 18.9 mU/L (7 ng/mL) as normal, with an absolute deficiency defined as <6.75 mU/L (Heikens *et al.* 1998). Maciel *et al.* used a cutoff of <7 ng/mL for GHD (Maciel *et al.* 2021), and Popovic *et al.* set stricter criteria, defining GHD as a peak GH < 3 μg/L (<3 ng/mL) in ITT or <10 μg/L in the growth hormone-releasing hormone (GHRH) combined with growth hormone-releasing peptide-6 (GHRP-6) test (Popovic *et al.* 2002). Shalet *et al.* also used a peak GH < 20 mU/L as a cutoff for GHD (Shalet *et al.* 1977). van Iersel *et al.* diagnosed GHD based on a history of dynamic testing or insulin-like growth factor 1 (IGF-1) level lower than −2 standard deviations from normal values (van Iersel *et al.* 2019). Baunsgaard *et al.* utilized either ITT or GHRH-arginine tests to diagnose GHD but did not provide further details on specific cutoffs (Baunsgaard *et al.* 2022). For consistency in the subgroup analysis, we used 3 and 7 ng/mL cutoffs. Where ng/mL units were not provided by authors, we used the commonly accepted conversion of 1 mU/L ≈ 0.33 ng/mL to convert from mU/L to ng/mL (Pokrajac *et al.* 2007).

Two studies specified the GH assay used for GH level measurement. Harrop *et al.* reported using the International Reference Preparation (IRP) 66/217 for measuring plasma GH, while Shalet *et al.* used the Medical Research Council (MRC) standard 66/217.

Six studies were conducted in Europe (Harrop *et al.* 1976, Shalet *et al.* 1977, Heikens *et al.* 1998, Popovic *et al.* 2002, Maciel *et al.* 2021, Baunsgaard *et al.* 2022), while one was done in North America (van Iersel *et al.* 2019). Four studies evaluated GHD at age ≥18 years in all patients (Harrop *et al.* 1976, Shalet *et al.* 1977, Heikens *et al.* 1998, Baunsgaard *et al.* 2022), one study evaluated GHD at age ≥18 years in 19 out of 22 patients (Popovic *et al.* 2002), and two studies did not report the number of patients evaluated for GHD at age ≥18 years.

### Incidence of GHD in patients who received cranial irradiation

**Figure 2:**
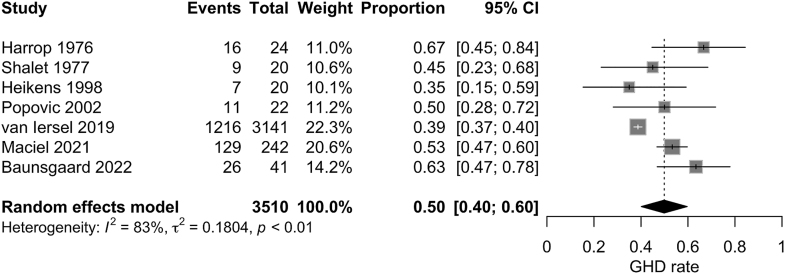
Forest plot of the incidence of adult GHD in patients with pediatric brain cancer who received cranial irradiation.

The meta-analysis of the entire patient group from all seven studies, regardless of age at the time of treatment, showed that patients who received cranial irradiation for brain tumors had a pooled incidence of developing GHD of 50% (95% CI 40–60%). The statistical heterogeneity was high (*I*^2^ = 83%) (Fig. 2). Egger’s test did not suggest an evidence of publication bias (*P* = 0.087).

### Incidence of adult GHD in patients who received cranial irradiation in childhood

**Figure 3:**
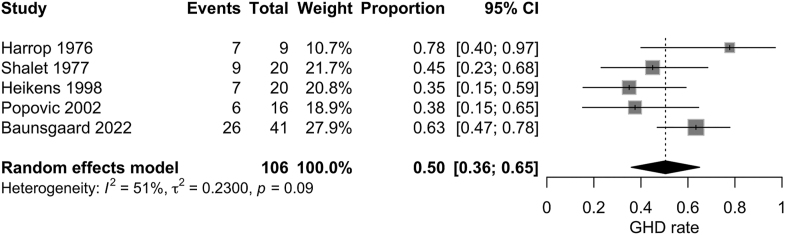
Sensitivity analysis of the incidence of adult GHD in patients with pediatric brain cancer who received cranial irradiation using studies that evaluated GHD at age of more than 18 years.

A sensitivity analysis of studies that determined GHD at age ≥18 years in patients who underwent cranial irradiation in childhood showed the same pooled incidence of 50% (95% CI 36–65%, *I*^2^ = 51%) (Fig. 3).

### Subgroup analyses

**Figure 4:**
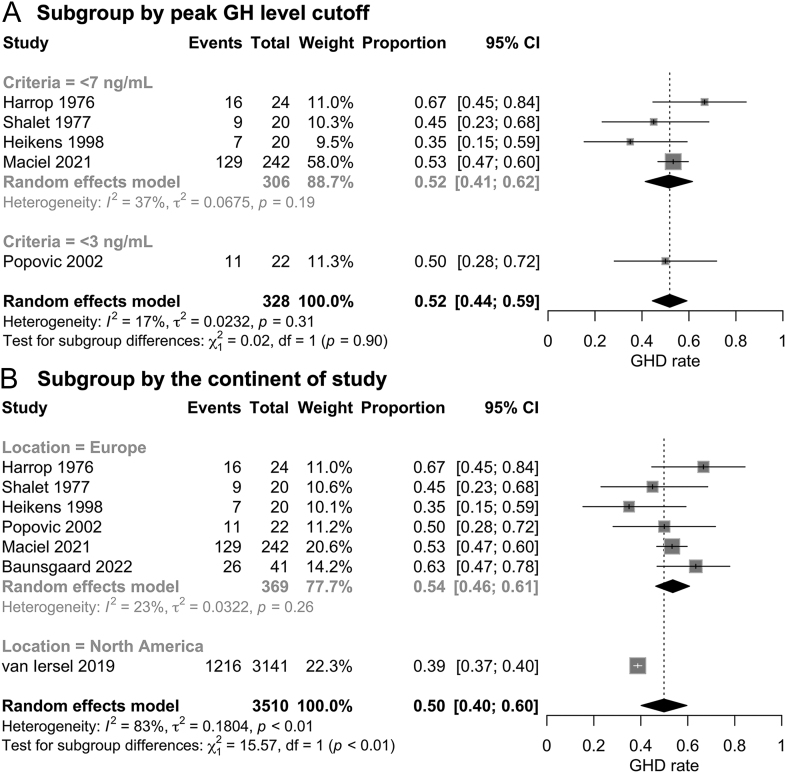
Subgroup analysis of the incidence of adult GHD in patients with pediatric brain cancer who received cranial irradiation (A) by the peak GH level cutoff, and (B) by the continent of the study.

Two subgroup analyses by peak GH level cutoff and the continent of study were performed. A peak GH level cutoff of <7 ng/mL did not result in a significantly higher rate of GHD compared to when the cutoff of <3 ng/mL was used (52 vs 50%, *P* = 0.90) (Fig. 4A). However, patients in the study from North America showed a lower incidence of GHD than those from Europe (39 vs 54%, *P* < 0.01) (Fig. 4B).

### Association between peak GH level and clinical factors

**Figure 5:**
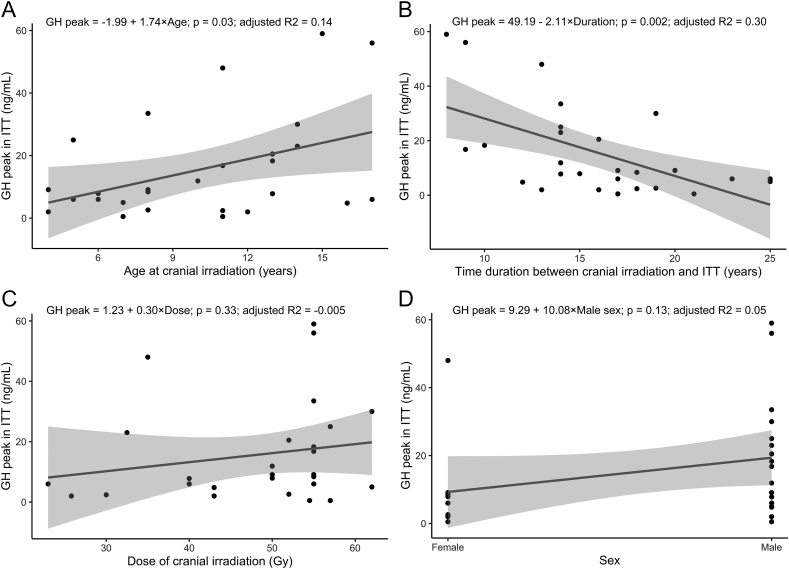
Univariate linear regression between peak GH level in ITT and (A) age at cranial irradiation, (B) time duration between cranial irradiation and ITT, (C) cranial irradiation dose, and (D) sex.

Four studies reported individual-level data; however, two studies (Shalet *et al.* 1977, Popovic *et al.* 2002) did not report total cranial irradiation dose, leaving two eligible studies with 29 patients for regression analysis. Of note, two patients were concomitantly receiving GH substitution therapy and were excluded, ultimately leaving 27 patients for final individual-level regression analysis (Harrop *et al.* 1976, Heikens *et al.* 1998) to further determine the factors associated with peak GH level. The parameters and associated values for the models are reported in Table 1. Age at cranial irradiation was positively associated with the peak GH level from ITT (estimate 1.74 ng/mL, *P* = 0.03, adjusted *R*^2^ = 0.14), while the time duration between cranial irradiation and ITT was negatively associated with the peak GH level (estimate −2.11 ng/mL, *P* = 0.0023, adjusted *R*^2^ = 0.30). Cranial irradiation dose and sex did not correlate with the peak GH level (Fig. 5A, B, C, D). However, only the time duration between cranial irradiation and ITT was associated after multivariate adjustment by age at cranial irradiation, cranial irradiation dose, and sex (estimate of the time duration −1.65 ng/mL, *P* = 0.04, adjusted *R*^2^ = 0.28).

**Table 1:** Multivariate linear regression of growth hormone peak level and clinical factors.

Model	Estimate	Standard error	*P*-value	Adjusted *R*^2^
**Age at cranial irradiation + time duration between cranial irradiation and ITT + cranial irradiation dose + male sex**				
Intercept	22.84	23.22	0.34	
Age at cranial irradiation	0.64	0.85	0.46	
Time duration between cranial irradiation and ITT	−1.65	0.77	0.04	
Cranial irradiation dose	0.20	0.27	0.48	
Male sex	4.86	6.18	0.44	0.28

Abbreviations: ITT, insulin tolerance test. Model adjusted for age at cranial irradiation, sex, time duration between cranial irradiation and ITT, and cranial irradiation dose.

## Discussion

This systematic review and meta-analysis included data from 3,489 patients who underwent cranial irradiation for brain tumors and revealed that approximately 50% would eventually develop GHD (95% CI 40–60%, *I*^2^ = 83%). GHD is known to be the most prevalent and earliest endocrinopathy to develop after the treatment of brain tumors due to the high susceptibility of somatotrophs to radiation. The GHD prevalence among included studies varied between 39 and 78%. The definition of GHD differed between studies, which can affect the reported prevalence. In one study, either ITT or the GHRH-arginine test was used for the GHD diagnosis (Baunsgaard *et al.* 2022), in another, an IGF-1 level lower than −2 standard deviations (SD) from normal values was used (van Iersel *et al.* 2019). Although IGF-1 can be helpful in identifying GHD in individuals with established hypopituitarism, its sensitivity is limited in diagnosing isolated GHD. It is particularly unclear if that is a reliable tool in irradiated populations. As a result, this could have led to some missed cases and to a lower overall estimate of GHD prevalence. In the rest, ITT was utilized for the diagnosis of GHD; however, different peak GH level cutoffs, as well as different measurement units of GH, were implemented.

It is important to mention that the GH peak cutoff limit for the GHD definition is still arbitrary. Per the Growth Hormone Research Society, GHD in adults is confirmatory if peak GH in ITT is <5 μg/L (Growth Hormone Research Society 1998). Endocrine Society Guidelines for the evaluation of GHD in adults have broader criteria for the GHD diagnosis. The cutoff for the peak GH in ITT of <5.1 μg/L and for the GHRH-arginine test of <4.1 μg/L is considered suggestive of GHD. They also suggest that the presence of deficiencies in three or more pituitary axes is sufficient for the GHD diagnosis, and provocative testing can be optional (Molitch *et al.* 2011). However, in this study, subgroup analysis based on the GH peak level did not show differences between different cutoffs. In addition, there is significant variability in GH measurement between each assay. This could pose a challenge when determining the common cutoff limits to interpret the results from different assays.

Another factor that may play a role in the variability of the prevalence of GHD is the modality, fractionation, and dosage of radiation therapy administration. The radiation dose ranged between 12 and 60 Gy. It is known that a higher cumulative radiation dose is associated with a higher incidence of GHD and other HP axis failures (Toogood 2004). The biological effective dose was also known to correlate with a higher risk of GHD (Schmiegelow *et al.* 2000*a*). A radiation dose divided into small fractions and administered over a longer period of time was less likely to cause GHD; however, the data were conflicting regarding whether a larger fraction size increases the risk of GHD (Shalet *et al.* 1979, Brauner *et al.* 1993, Thomas *et al.* 1993, Brauner *et al.* 1997). We did not perform a detailed analysis based on the type of radiation because it was not feasible, as this information was not uniformly available across studies. Future studies with larger, well-characterized cohorts will be necessary to explore whether specific radiation modalities confer a higher risk for GHD. Similarly, we were unable to analyze the relationship between the type of brain tumor and the incidence of GHD.

In the studies included in this meta-analysis, higher doses of CRT and a younger age at the time of treatment were suggestive of the development of GHD (Harrop *et al.* 1976, Shalet *et al.* 1977, Heikens *et al.* 1998). Shalet *et al.* showed that the damage to the HP axis predominantly depends on the radiation dose (Shalet *et al.* 1977), and impairment in response increases with time after radiation, which was suggested in their earlier study (Shalet *et al.* 1975). A radiation dose of at least 2,500–2,900 rads is required to affect GH secretion (Shalet *et al.* 1976, Shalet *et al.* 1977). However, GHD has been observed in patients after total body irradiation before bone marrow transplantation, where doses can be as low as 8–11 Gy. Heikens *et al.* showed that GHD was associated with younger age at the time of treatment in patients treated for medulloblastoma (Heikens *et al.* 1998). It was postulated that, due to slowly progressing radiation-induced changes, HP deficiencies become more apparent with longer follow-up time, which was shown in several studies (Shalet *et al.* 1975, Heikens *et al.* 2000, Mulder *et al.* 2009). None of the studies included in this meta-analysis suggested a longer time since treatment to be the determinant of GHD. However, univariate linear regression of combined data from 27 patients of interest from two studies showed that the time duration between radiation and GH test was associated with GHD, suggesting slowly developing radiation-induced changes.

The degree of the contribution of chemotherapeutic agents to GHD in particular, and damage to the HP axis in general, remains controversial (Crowne *et al.* 2015) and was shown only in small case series (Voorhess *et al.* 1986, Birkebaek *et al.* 1998, Rose *et al.* 2004). Van Iersel *et al.* demonstrated that alkylating agents may facilitate the development of GHD, but not play an independent role (van Iersel *et al.* 2019). Conversely, they reported that the link between GHD and intrathecal chemotherapy remained significant only in individuals not exposed to HP radiotherapy. This was limited by the inability to determine whether alkylating agents and intrathecal chemotherapy resulted in toxicity to the HP axis or an enhancement of the damage caused by radiotherapy.

Our analysis has some limitations that are worth noting. First, the heterogeneity of the included studies, including the study design and patient selection, differed between studies. Some of them did not report the detailed patient selection criteria (Shalet *et al.* 1977, Popovic *et al.* 2002), while some required patients to survive past the 5-year mark (Heikens *et al.* 1998, Baunsgaard *et al.* 2022) to be included in the study. Second, only four out of seven included studies reported patient-level data to evaluate the potential associated factors of developing GHD. Only two of these studies featured a more homogeneous patient population and consistent radiation regimen, allowing for a more reliable comparison within these limited parameters. Finally, we were unable to take into consideration the fractionation of the doses of cranial irradiation due to the lack of data.

In conclusion, this meta-analysis showed that the incidence of GHD in patients after treatment for brain tumors in adult childhood brain tumor survivors is approximately 50%. The main risk factor is the duration of the follow-up. While the link between cranial irradiation and GHD is well established, this review underscores the importance of long-term monitoring, as GHD can manifest many years after treatment. Given the high prevalence of GHD, GH function should be assessed in the follow-up for all patients who underwent cranial irradiation for CNS tumors in childhood. These patients would benefit from monitoring for signs of GHD to allow timely diagnosis and management. Studies with a more robust design are needed to better evaluate the prevalence and risk factors of GHD in adult childhood brain tumor survivors, with uniform methods of GHD detection and a sufficient follow-up period.

## Supplementary materials



## Declaration of interest

Dr Betty M Drees has funding from Abbott Diabetes Care (education grant), the William G McGowan Charitable Fund (research), and Viridian Therapeutics, Inc. (Research). Other authors have no conflicts of interest to declare. All co-authors have seen and agree with the contents of the manuscript, and there is no financial interest to report.

## Funding

This work did not receive any specific grant from any funding agency in the public, commercial, or not-for-profit sector.

## Author contribution statement

TT designed the project. TT and BP performed the literature search, examined the eligibility of the studies, assessed the quality of included studies, and drafted the manuscript. BP processed the data, performed analysis, and designed the figures and table. MGP conceived the original idea. CF contributed to search terms, optimized search strategies, and suggested journals for submission. SSR provided expertise on radiation-related aspects. BMD provided overall supervision of the project, addressed queries, critically revised the manuscript for scientific accuracy and content, and guided the project.
