# Supplementary material for: Prevalence of growth hormone deficiency in brain tumor survivors: a systematic review and meta-analysis
Source: Endocr Oncol. 2025 Jul 8;5(1):e250025. doi: 10.1530/EO-25-0025 (PMC12243098; doi:10.1530/EO-25-0025)
Supplement: Supplementary file 1 [file supplementary_materials.pdf]

Supplementary Material

Supplementary Data 1 Search Strategy

Embase database

1. 'conformal radiotherapy'/exp OR 'conformal radiotherapy'
2. 'external beam radiotherapy'/exp OR 'external beam radiotherapy'
3. 'intensity modulated radiation therapy'/exp OR 'intensity modulated radiation therapy'
4. 'proton therapy'/exp OR 'proton therapy'
5. 'cranial radiation therapy'/exp OR 'cranial radiation therapy'
6. 'whole brain radiotherapy'/exp OR 'whole brain radiotherapy'
7. 'whole brain irradiation'/exp OR 'whole brain irradiation'
8. 'whole brain radiation'/exp OR 'whole brain radiation'
9. 'neutron capture therapy'/exp OR 'neutron capture therapy'
10. 'skull irradiation'/exp OR 'skull irradiation'
11. 'hypophysis'/exp OR 'hypophysis'
12. 'hypophysis disease'/exp OR 'hypophysis disease'
13. 'hypophysis hormone'/exp OR 'hypophysis hormone'
14. 'adenohypophysis hormone'/exp OR 'adenohypophysis hormone'
15. 'hypopituitarism'/exp OR 'hypopituitarism'
16. 'growth hormone'/exp OR 'growth hormone'
17. 'growth hormone deficiency'/exp OR 'growth hormone deficiency'
18. 'growth hormone secreting cell'/exp OR 'growth hormone secreting cell'
19. 'hypophysis function'/exp OR 'hypophysis function'
20. 'brain tumor'/exp OR 'brain tumor'
21. 'brain cancer'/exp OR 'brain cancer'
22. 'intracranial tumor'/exp OR 'intracranial tumor'
23. 'central nervous system cancer'/exp OR 'central nervous system cancer'
24. 'central nervous system tumor'/exp OR 'central nervous system tumor'
25. 'skull tumor'/exp OR 'skull tumor'
26. 'skull cancer'/exp OR 'skull cancer'
27. 'posterior cranial fossa tumor'/exp OR 'posterior cranial fossa tumor'
28. 'middle cranial fossa tumor'/exp OR 'middle cranial fossa tumor'
29. #1 OR #2 OR #3 OR #4 OR #5 OR #6 OR #7 OR #8 OR #9 OR #10
30. #11 OR #12 OR #13 OR #14 OR #15 OR #16 OR #17 OR #18 OR #19
31. #20 OR #21 OR #22 OR #23 OR #24 OR #25 OR #26 OR #27 OR #28
32. #29 AND #30 AND #31

Medline database

1. conformal radiotherapy.mp. or exp Radiotherapy, Conformal/
2. external beam radiotherapy.mp.
3. exp Radiotherapy, Intensity-Modulated/ or exp Radiotherapy Planning, Computer-Assisted/ or intensity modulated radiation therapy.mp.
4. proton therapy.mp. or exp Proton Therapy/
5. cranial radiation therapy.mp. or exp Cranial Irradiation/

6. whole brain radiotherapy.mp.
7. whole brain irradiation.mp.
8. neutron capture therapy.mp. or exp Boron Neutron Capture Therapy/ or exp Neutron Capture Therapy/
9. skull irradiation.mp.
10. pituitary disease.mp. or exp Pituitary Diseases/
11. pituitary hormone.mp. or exp Pituitary Hormones/
12. hypopituitarism.mp. or exp Hypopituitarism/
13. growth hormone.mp. or exp Growth Hormone/
14. exp Human Growth Hormone/ or growth hormone deficiency.mp.
15. somatotroph.mp. or exp Somatotrophs/
16. brain tumor.mp. or exp Brain Neoplasms/
17. intracranial tumor.mp.
18. exp Central Nervous System Neoplasms/ or central nervous system cancer.mp.
19. exp Skull Neoplasms/ or exp Skull Base Neoplasms/ or skull cancer.mp.
20. exp Infratentorial Neoplasms/ or posterior cranial fossa tumor.mp. or exp Cranial Fossa, Posterior/
21. 1 or 2 or 3 or 4 or 5 or 6 or 7 or 8 or 9
22. 10 or 11 or 12 or 13 or 14 or 15
23. 16 or 17 or 18 or 19 or 20

21 and 22 and 23

Supplementary Data 2 – PRISMA Checklist

Section/topic	#	Checklist item	Reported on page #
**TITLE**			
Title	1	Identify the report as a systematic review, meta-analysis, or both.	
**ABSTRACT**			
Structured summary	2	Provide a structured summary including, as applicable: background; objectives; data sources; study eligibility criteria, participants, and interventions; study appraisal and synthesis methods; results; limitations; conclusions and implications of key findings; systematic review registration number.	
**INTRODUCTION**			
Rationale	3	Describe the rationale for the review in the context of what is already known.	
Objectives	4	Provide an explicit statement of questions being addressed with reference to participants, interventions, comparisons, outcomes, and study design (PICOS).	
**METHODS**			
Protocol and registration	5	Indicate if a review protocol exists, if and where it can be accessed (e.g., Web address), and, if available, provide registration information including registration number.	
Eligibility criteria	6	Specify study characteristics (e.g., PICOS, length of follow-up) and report characteristics (e.g., years considered, language, publication status) used as criteria for eligibility, giving rationale.	
Information sources	7	Describe all information sources (e.g., databases with dates of coverage, contact with study authors to identify additional studies) in the search and date last searched.	
Search	8	Present full electronic search strategy for at least one database, including any limits used, such that it could be repeated.	Supp.1
Study selection	9	State the process for selecting studies (i.e., screening, eligibility, included in systematic review, and, if applicable, included in the meta-analysis).	
Data collection process	10	Describe method of data extraction from reports (e.g., piloted forms, independently, in duplicate) and any processes for obtaining and confirming data from investigators.	
Data items	11	List and define all variables for which data were sought (e.g., PICOS, funding sources) and any assumptions and simplifications made.	

Risk of bias in individual studies	12	Describe methods used for assessing risk of bias of individual studies (including specification of whether this was done at the study or outcome level), and how this information is to be used in any data synthesis.	
Summary measures	13	State the principal summary measures (e.g., risk ratio, difference in means).	
Section/topic	#	Checklist item	Reported on page #
Synthesis of results	14	Describe the methods of handling data and combining results of studies, if done, including measures of consistency (e.g., I^2) for each meta-analysis.	
Risk of bias across studies	15	Specify any assessment of risk of bias that may affect the cumulative evidence (e.g., publication bias, selective reporting within studies).	
Additional analyses	16	Describe methods of additional analyses (e.g., sensitivity or subgroup analyses, meta-regression), if done, indicating which were pre-specified.	
**RESULTS**			
Study selection	17	Give numbers of studies screened, assessed for eligibility, and included in the review, with reasons for exclusions at each stage, ideally with a flow diagram.	
Study characteristics	18	For each study, present characteristics for which data were extracted (e.g., study size, PICOS, follow-up period) and provide the citations.	
Risk of bias within studies	19	Present data on risk of bias of each study and, if available, any outcome level assessment (see item 12).	
Results of individual studies	20	For all outcomes considered (benefits or harms), present, for each study: (a) simple summary data for each intervention group (b) effect estimates and confidence intervals, ideally with a forest plot.	
Synthesis of results	21	Present results of each meta-analysis done, including confidence intervals and measures of consistency.	
Risk of bias across studies	22	Present results of any assessment of risk of bias across studies (see Item 15).	
Additional analysis	23	Give results of additional analyses, if done (e.g., sensitivity or subgroup analyses, meta-regression [see Item 16]).	Fig. 3A-B
**DISCUSSION**			
Summary of evidence	24	Summarize the main findings including the strength of evidence for each main outcome; consider their relevance to key groups (e.g., healthcare providers, users, and policy makers).	

Limitations	25	Discuss limitations at study and outcome level (e.g., risk of bias), and at review-level (e.g., incomplete retrieval of identified research, reporting bias).	
Conclusions	26	Provide a general interpretation of the results in the context of other evidence, and implications for future research.	
**FUNDING**			
Funding	27	Describe sources of funding for the systematic review and other support (e.g., supply of data); role of funders for the systematic review.	

From: Moher D, Liberati A, Tetzlaff J, Altman DG, The PRISMA Group (2009). Preferred Reporting Items for Systematic Reviews and Meta-Analyses: The PRISMA Statement. PLoS Med 6(7): e1000097. doi:10.1371/journal.pmed1000097

For more information, visit: **www.prisma-statement.org**.

Supplementary Material

Table S1 Characteristics of the included studies.

**Author**	Baunsgaard et al.	Harrop et al.	Heikens et al.	Maciel et al.	Popovic et al.	Shalet et al.	van Iersel et al.
**Country**	Denmark	UK	Netherlands	Portugal	Spain	UK	USA
**Study design**	Prospective and retrospective	Retrospective	Prospective	Retrospective	Prospective	Retrospective	Retrospective
**Year of publication**	2022	1976	1998	2021	2002	1977	2019
**Total number of participants**	41	24	20	242	22	20	3141
**Number or % of GHD patients**	26	16	7	129	11	9	38.7%

**Recruitment of participants**	Five-years survivors from two health regions in Denmark, treated for a brain tumor in childhood with CNS irradiation in 1997-2015. Irradiated survivors, where a GH test at final height had not been done, were invited.	Patients in whom extrasellar lesion had been diagnosed in the past. 17 - received a course of external CI, 7 - did not get CI, and only had surgery.	The study was performed in subjects treated for medulloblastoma during childhood at the Emma Kinderziekenhuis, Amsterdam, The Netherlands. Subjects were eligible if it was more than 5 years after the cessation of treatment and if they were older than 18 years at the time of the investigation. Exclusion criteria were recent seizures, symptomatic ischemic	All patients with past history of childhood brain tumor between 1994-2018.	Not mentioned.	Not mentioned.	Patients were recruited from SJLIFE study from St. Jude Children's Research Hospital from 2007-2016.

			heart disease and pregnancy. Two mentally retarded patients were excluded because no written informed consent could be obtained.				
**Type of tumor**	Not specified	Astrocytoma - 9; meningioma - 4; angioma - 1; ependymoma - 1; pinealoma - 1; thalamic (no biopsy) - 1; III	Medulloblastoma only	Various primary brain tumors	Medulloblastoma - 12; pineal tumor - 4; ganglioglioma - 3; meningioma anaplasticum - 1; ependymoma - 2.	Glioma - 10; medulloblasto ma - 8; reticulosarcom a - 1; N/A - 1.	Various primary brain tumors

		ventricle (no biopsy) - 4; suprasellar (no biopsy) - 2; cerebellar (no biopsy) - 1.					
**Dose of CI, Gy**	12-16 Gy	2300-5200 rads	35 +-2.6 Gy with a boost to the posterior fossa of 18 +-3.7 Gy. The irradiation doses to the craniospinal axis were fractionated as follows: fractions of 1.5 Gy to the cranium, fractions of 1.5 Gy on the spinal axis and boost fractionation doses between 1.5 and 1.8 Gy.	Median 54 Gy (24-60)	Mean 52.8 +/- 1.4 Gy	2000-5000 rads	1->30 Gy

**N of patients, who received chemotherapy**	Not mentioned	Not mentioned	13	184	Not mentioned	0	2676
**Age at CI, years**	Not mentioned. Median 14.8 years (5.1-23.4) since diagnosis	Median 28.5 (4-63)	Median 8 (4-17)	Median 6.7 (0-18)	15.6 +/- 1.3	Not mentioned. Mean 14.2 (8-32) since irradiation	Median 6.8 (0-18)
**Age at GHD test, years**	Median 21.7 (15.1-33.8)	Median 34.5 (20-72)	Median 25 (19-33)	Median 12.1 (3.6-29.0)	23.2 +/- 1.4	Mean 24.2 (15-45)	Median 31.7 (7.5-65.1)
**GHD definition**	Either ITT or GHRH-arginine was used but no other details provided.	ITT: peak GH <20 mu/L. Glucagon test: peak GH <10 mu/L.	ITT: peak GH < 18.9 mU/l (< 7 ng/ml). An absolute GHD: peak < 6.75 mU/l and a subnormal response as a peak response between 6.75 and 18.9	ITT: peak GH < 7 ng/mL.	ITT: peak GH < 3 μg/L . GHRH+GHRP6: peak GH < than 10 μg/L	ITT: peak GH < 20 mU/L	IGF-1 level lower than -2 SD of normal values or past history of GHD based on dynamic testing

			mU/l.				
**Percentage of male**	46.3%	46%	70%	55%	63.6%	60%	52.1%
**Comorbidities**	Not mentioned	Not mentioned	Not mentioned	Not mentioned	Not mentioned	Not mentioned	Stroke 5.1%, seizure 12.2%
**Other HP hormones deficiencies**	17%, 15%, and 15% had 2, 3, and 4 hormonal deficiencies respectively	Hypogonadsim - 2; hypocortisolism - 3; hypothyroidism - 1	Central hypogonadism -3; subclinical hypothyroidism - 7, marginal central hypothyroidism -1; primary hypothyroidism -1; hypocortisolism - 1	Hypothyroidism - 92; hypogonadism - 61; ACTH deficiency - 25; diabetes insipidus - 29	Hyperprolactinemia - 1	Hypothyroidism - 3	Hypothyroidism - 6.1%; hypogonadism - 9.7%; adrenal insufficiency - 3.6%
**Newcastle-Ottawa score: selection, comparability, outcome**	3, 0, 1	3, 0, 2	3, 0, 1	3, 0, 1	3, 0, 2	3, 0, 1	3, 2, 2

Abbreviations: CI - cranial irradiation; CNS - central nervous system; GH - growth hormone; GHD - growth hormone deficiency; GHRH - growth hormone-releasing hormone; GHRP6 - growth hormone-releasing peptide-6; Gy - Gray; HP - hypothalamic-pituitary; ITT - insulin tolerance test; N/A - not applicable.

References:

BAUNSGAARD, M. M., HELLIGSOE, A. S. L., HENRIKSEN, L. T., MIKKELSEN, T. S., CALLESEN, M., WEBER, B., HASLE, H. & BIRKEBAK, N. 2022. Growth hormone deficiency in adult survivors of childhood brain tumors treated with irradiation. Hormone Research in Paediatrics, 95, 96-97.

HARROP, J. S., DAVIES, T. J., CAPRA, L. G. & MARKS, V. 1976. Hypothalamic pituitary function following successful treatment of intracranial tumours. Clinical Endocrinology, 5, 313-321.

HEIKENS, J., MICHIELS, E. M., BEHRENDT, H., ENDERT, E., BAKKER, P. J. & FLIERS, E. 1998. Long-term neuro-endocrine sequelae after treatment for childhood medulloblastoma. European journal of cancer (Oxford, England : 1990), 34, 1592-7.

MACIEL, J., DIAS, D., CAVACO, D., DONATO, S., PEREIRA, M. C. & SIMÕES-PEREIRA, J. 2021. Growth hormone deficiency and other endocrinopathies after childhood brain tumors: results from a close follow-up in a cohort of 242 patients. Journal of Endocrinological Investigation, 44, 2367-2374.

POPOVIC, V., PEKIC, S., GOLUBICIC, I., DOKNIC, M., DIEGUEZ, C. & CASANUEVA, F. F. 2002. The impact of cranial irradiation on GH responsiveness to GHRH plus GH-releasing peptide-6. Journal of Clinical Endocrinology and Metabolism, 87, 2095-2099.

SHALET, S. M., BEARDWELL, C. G., MACFARLANE, I. A., JONES, P. H. & PEARSON, D. 1977. Endocrine morbidity in adults treated with cerebral irradiation for brain tumours during childhood. Acta endocrinologica, 84, 673-80.

VAN IERSEL, L., LI, Z., SRIVASTAVA, D. K., BRINKMAN, T. M., BJORNARD, K. L., WILSON, C. L., GREEN, D. M., MERCHANT, T. E., PUI, C.-H., HOWELL, R. M., SMITH, S. A., ARMSTRONG, G. T., HUDSON, M. M., ROBISON, L. L., NESS, K. K., GAJJAR, A., KRULL, K. R., SKLAR, C. A., VAN SANTEN, H. M. & CHEMAITILLY, W. 2019. Hypothalamic-Pituitary Disorders in Childhood Cancer Survivors: Prevalence, Risk Factors and Long-Term Health Outcomes. The Journal of clinical endocrinology and metabolism, 104, 6101-6115.
